# Severe bradycardia after amivantamab administration in a patient with epidermal growth factor receptor-positive non-small cell lung cancer: A case report

**DOI:** 10.20407/fmj.2026-003

**Published:** 2026-05-14

**Authors:** Shoko Kamenosono, Aki Ikeda, Hideaki Takahashi, Toshikazu Watanabe, Tetsunari Hase, Nanami Kiryu, Shin Hasegawa, Yoshiko Shigeyasu, Tomohide Souma, Tomoya Horiguchi, Yasuhiro Goto, Naozumi Hashimoto, Kazuyoshi Imaizumi

**Affiliations:** Department of Respiratory Medicine, Fujita Health University, School of Medicine, Toyoake, Aichi, Japan

**Keywords:** Amivantamab, Non-small cell lung cancer, Bradycardia, Infusion-related reactions

## Abstract

Amivantamab, a bispecific antibody targeting epidermal growth factor receptor (EGFR) and mesenchymal–epithelial transition factor, has shown clinical efficacy in patients with *EGFR*-mutated non-small cell lung cancer. Although infusion-related reactions (IRRs) are common adverse events associated with this drug, cardiac events such as bradycardia have rarely been reported. We report the case of a 53-year-old Japanese man with advanced *EGFR* exon 19-deleted lung adenocarcinoma who was treated with amivantamab plus lazertinib as eighth-line therapy. On the first day, he experienced dyspnea, chills, and headache 30 min after amivantamab administration, so the infusion flow was reduced to 25 mL/h. After the symptoms resolved, the flow rate was set to 50 mL/h. On the second day, 2 h after initiating amivantamab infusion (700 mg) at 50 mL/h, his heart rate decreased to <50 beats per minute (bpm) and subsequently to <40 bpm. His blood pressure remained stable and he had no symptoms. The infusion was interrupted and intravenous methylprednisolone, chlorpheniramine, and atropine were administered. Bradycardia persisted for approximately 24 h but gradually resolved. On rechallenge with amivantamab 10 days later, bradycardia did not recur, suggesting that bradycardia is an IRR. Clinicians should be aware of this potential reaction and closely monitor cardiac rhythm during and after amivantamab infusion.

## Introduction

Amivantamab is a bispecific antibody targeting epidermal growth factor receptor (EGFR) and mesenchymal–epithelial transition factor (MET) that causes antibody-dependent cellular cytotoxicity, ligand blocking, receptor degradation, and trogocytosis.^1^ The combination of amivantamab and lazertinib, approved for the treatment of *EGFR*-mutated advanced non-small cell lung cancer (NSCLC) based on the results of the MARIPOSA trial,^2^ represents a standard therapy. Cardiac adverse events associated with amivantamab are rare but may occur as infusion-related reactions (IRRs).^3^ Here, we report a novel case of severe bradycardia following amivantamab administration.

## Case report

A 53-year-old Japanese man was referred to our department after an annual check-up chest X-ray showed right pleural effusion. He had a history of hypothyroidism, treated with daily levothyroxine, and no history of arrhythmia. There was no family history of cancer or arrhythmia.

After thoracentesis, the patient was diagnosed with stage IVA (cT1bN0M1a) lung adenocarcinoma harboring an *EGFR* exon 19 deletion. Despite several lines of treatment, including osimertinib, carboplatin, paclitaxel, bevacizumab, atezolizumab, pemetrexed, docetaxel, ramucirumab, gemcitabine, and nab-paclitaxel, the disease progressed, so combination therapy with amivantamab and lazertinib was initiated as eighth-line therapy. Laboratory tests performed before treatment revealed no clinically significant abnormalities (Table 1). His Eastern Cooperative Oncology Group performance status was 0 and the baseline electrocardiogram (ECG) showed no abnormalities. He had a heart rate (HR) of 68 beats per minute (bpm) (Figure 1a).

On day 1 after premedication with chlorpheniramine maleate, dexamethasone, and famotidine, amivantamab was administered at a dose of 350 mg in 250 mL of saline at a rate of 50 mL/h (Figure 2). Lazertinib (240 mg once orally) was administered daily. Approximately 30 min after the administration of amivantamab, he complained of dyspnea, chills, and headache; therefore, the infusion flow was reduced to 25 mL/h because of a suspected IRR. His symptoms resolved after 2 h, so the flow rate was again set at 50 mL/h. On day 2, amivantamab was administered at a dose of 700 mg in 250 mL of saline at a rate of 50 mL/h. Two hours after the start of the infusion, his HR decreased to <50 bpm, although his blood pressure (BP) remained stable and no symptoms were observed. After another hour, his HR decreased to <40 bpm, although his BP was sustained (Figures 2, 3). The infusion was interrupted as a suspected IRR caused by amivantamab, and methylprednisolone (40 mg) and chlorpheniramine maleate (5 mg) were administered intravenously. Because bradycardia persisted, amivantamab infusion was discontinued and atropine (0.5 mg) was administered for his slow HR. Transient hypertension following atropine required temporary nicardipine (1 mg bolus followed by 0.5 mg/h IV) use, but his BP soon stabilized and no symptoms developed, despite severe bradycardia. Because the patient also presented with hypoxia (an oxygen saturation of 91% in room air), oxygen was administered temporarily. The following day, bradycardia (HR <50 bpm) persisted (Figure 1b) but gradually resolved. Electrolyte levels were within normal limits (Table 1) and echocardiography showed no abnormalities.

After a risk–benefit discussion with the patient and cardiology consultation, amivantamab (350 mg) was administered 10 days later. No recurrence of bradycardia was observed, and treatment was performed as per the manufacturer’s instructions. Lazertinib therapy was continued without interruption.

## Discussion

IRRs are harmful reactions associated with the infusion of agents, particularly monoclonal antibodies.^4^ Amivantamab, an *EGFR*–*MET* bispecific antibody, causes IRRs in approximately 63% of patients.^2^ Typical symptoms include chills, fever, mild hypotension, dyspnea, and rash, which usually occur on day 1 of cycle 1.^5^ Regarding cardiac adverse events, several trials have reported tachycardia as an IRR in 3%–5% of cases.^6,7^ Moreover, an analysis of the U.S. FDA Adverse Event Reporting System identified nine and four cases of tachycardia and bradycardia, respectively, related to amivantamab;^3^ however, detailed clinical information was not available.

In our case, severe bradycardia occurred approximately 2 h after the start of amivantamab infusion on day 2. Although the patient received steroids and antihistamines for a suspected IRR, bradycardia (30–40 bpm) persisted, but promptly responded to atropine. We considered this event to represent an IRR for the following reasons: (1) bradycardia occurred without hypotension;^8^ (2) hypoxia without wheeze co-occurred with bradycardia;^8^ (3) previous reports have described bradycardia induced by monoclonal antibodies or even intravenous immunoglobulin as an IRR^9–12^ (Table 2); and (4) amivantamab was re-administered without the recurrence of bradycardia.^8^ To the best of our knowledge, this is the first report describing a potential association between amivantamab and bradycardia. Therefore, close ECG monitoring should be considered for patients receiving amivantamab infusions.

Although bradycardia occurred on day 2 of infusion in the present study, in the Phase I CHRYSALIS trial, almost all IRRs (98%) occurred with the first infusion, whereas only 4% of IRRs occurred with day 2 infusion.^13^ From a pharmacokinetic perspective, a rapid increase and high concentration of a monoclonal antibody in the serum is associated with rapid cytokine release, resulting in severe IRRs.^14^ This patient experienced an IRR on day 1 of the infusion; therefore, the rate was not increased at the end of this infusion (50 mL/h). On day 2, infusion with twice the initial concentration of amivantamab at the same infusion rate (50 mL/h) was performed, which may explain why an IRR also occurred on this day. Although the underlying mechanism remains unclear, it appears that if a patient experiences an IRR on day 1, the potential recurrence of IRRs on day 2 should be considered. The subcutaneous administration of amivantamab, which is associated with a slower increase and lower maximum concentration of this agent in the serum, was approved in Japan based on the PALOMA-3 trials, resulting in a lower incidence of IRRs.^6^

In conclusion, we encountered a case of bradycardia as an IRR after the administration of amivantamab. Although this is a rare event, amivantamab plus lazertinib is a standard treatment option for patients with *EGFR* mutation-positive NSCLC; therefore, physicians should consider and manage this rare adverse event during amivantamab infusion.

## Figures and Tables

**Figure 1:**
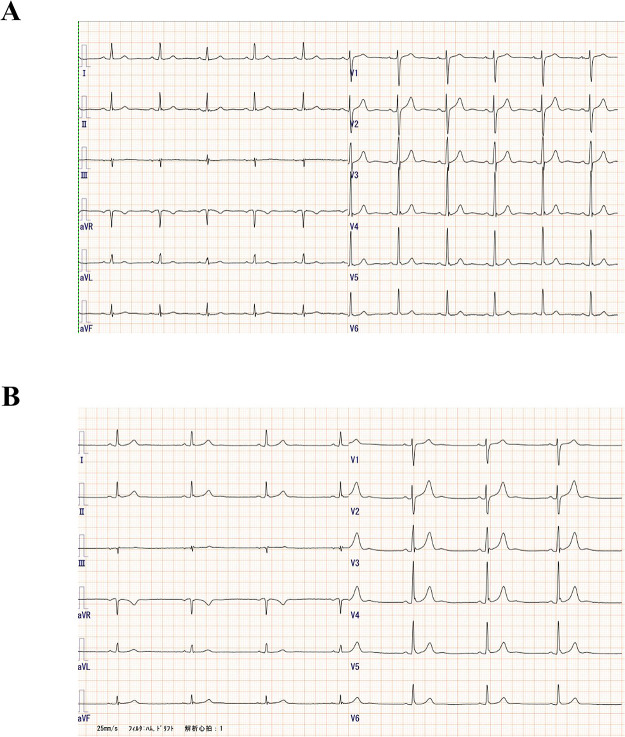
Changes in electrocardiogram (ECG) findings. ECGs recorded before the administration of amivantamab (a) and on day 3 (b). The heart rate and corrected QT were 68 bpm and 0.387 s (a) and 45 bpm and 0.382 s (b), respectively. Abbreviations: bpm, beats per minute.

**Figure 2:**
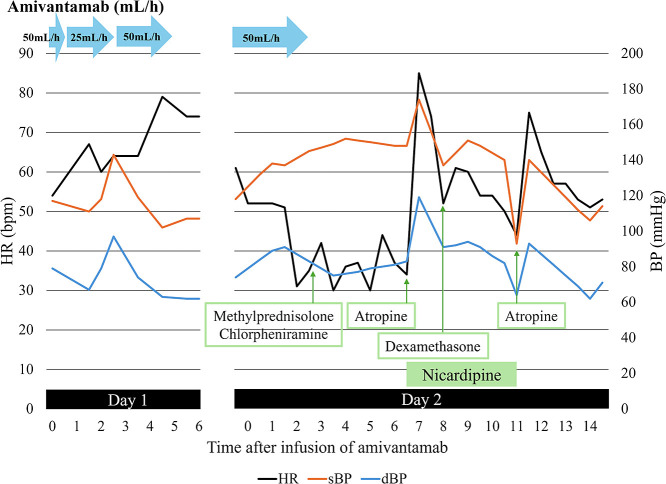
Time course of amivantamab administration. Abbreviations: sBP, systolic blood pressure; dBP, diastolic blood pressure; HR, heart rate.

**Figure 3:**
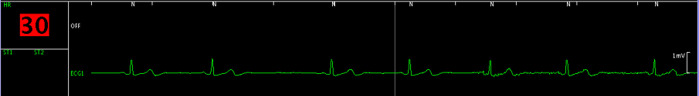
Electrocardiogram recorded using remote electrocardiographic monitoring 2 h after the administration of amivantamab on day 2.

**Table 1:** Laboratory results before treatment with amivantamab and lazertinib and after the occurrence of bradycardia

Laboratory parameter	Values before treatment	Values after bradycardia
White blood cell (10^9^/L)	15.4*	16.1*
Hemoglobin (g/dL)	11.3*	12.2*
Platelet count (10^9^/L)	293	232
Urea nitrogen (mg/dL)	17.0	21.1*
Creatinine (mg/dL)	0.96	1.12*
Sodium (mEq/L)	140	139
Potassium (mEq/L)	4.2	3.9
Bilirubin (mg/dL)	0.4	0.5
Alanine aminotransferase (U/L)	12	29
Aspartate aminotransferase (U/L)	16	16
Albumin (g/dL)	4.2	3.6*
C-reactive protein (mg/dL)	0.43*	3.35*

*indicates outside the normal range

**Table 2:** Summary of previous and current case reports on bradycardia as an infusion-related reaction.

Reference	Age (years)	Sex	Diseases	Medication	Time to onset	Baseline ECG	Other symptoms	Treatment	Re-administration
9	61	Male	Idiopathic thrombocytopenic purpura	Intravenous immunoglobulin	30 min	No apparent abnormality	Chest pain and shortness of breath	Atropine and dopamine	Terminated
10	56	Female	Diffuse large B cell lymphoma	Rituximab	2 days	AV Wenckebach	Syncope	Pacemaker implantation	Re-administration
11	86	Male	COVID-19	Bebtelovimab	30 min	Atrial fibrillation	Unresponsive with agonal respirations	Atropine	Terminated
12	71	Male	Colon cancer	Bevacizumab	N/A	No apparent abnormality	Chills, fever	Steroid and histamine antagonist	Re-administration with reduced infusion rate along with premedication with steroid and histamine antagonist without recurrence of bradycardia
Our case	53	Male	*EGFR* mutation-positive NSCLC	Amivantamab and lazertinib	2 h	No apparent abnormality	None	Steroid, histamine antagonist, and atropine	Re-administration without recurrence of bradycardia

Abbreviations: ECG, electrocardiogram; NSCLC, non-small cell lung cancer

## References

[1] Cho BC, Simi A, Sabari J, Vijayaraghavan S, Moores S, Spira A. Amivantamab, an epidermal growth factor receptor (EGFR) and mesenchymal-epithelial transition factor (MET) bispecific antibody, designed to enable multiple mechanisms of action and broad clinical applications. Clin Lung Cancer 2023; 24: 89–97. 36481319 10.1016/j.cllc.2022.11.004

[2] Cho BC, Lu S, Felip E, et al. Amivantamab plus lazertinib in previously untreated EGFR-mutated advanced NSCLC. N Engl J Med 2024; 391: 1486–1498. 38924756 10.1056/NEJMoa2403614

[3] Zhang J, Li W. Real-world pharmacovigilance analysis unveils the toxicity profile of amivantamab targeting EGFR exon 20 insertion mutations in non-small cell lung cancer. BMC Pulm Med 2025; 25: 63. 39915804 10.1186/s12890-025-03509-z PMC11800505

[4] Barroso A, Estevinho F, Hespanhol V, Teixeira E, Ramalho-Carvalho J, Araújo A. Management of infusion-related reactions in cancer therapy: strategies and challenges. ESMO Open 2024; 9: 102922. 38452439 10.1016/j.esmoop.2024.102922 PMC10937241

[5] Park K, Sabari JK, Haura EB, et al. Management of infusion-related reactions (IRRs) in patients receiving amivantamab in the CHRYSALIS study. Lung Cancer 2023; 178: 166–171. 36868177 10.1016/j.lungcan.2023.02.008

[6] Leighl NB, Akamatsu H, Lim SM, et al. Subcutaneous versus intravenous amivantamab, both in combination with lazertinib, in refractory EGFR-mutated NSCLC: primary results from the phase 3 paloma-3 study. J Clin Oncol 2024; 42: 3593–3605. 38857463 10.1200/JCO.24.01001 PMC11469630

[7] Spira AI, Paz-Ares L, Han JY, et al. Preventing infusion-related reactions with intravenous amivantamab: results from SKIPPirr, a phase 2 study. J Thorac Oncol 2025; 20: 809–816. 39864547 10.1016/j.jtho.2025.01.018

[8] Doessegger L, Banholzer ML. Clinical development methodology for infusion‐related reactions with monoclonal antibodies. Clin Transl Immunology 2015; 4: e39. 10.1038/cti.2015.14 PMC4524952 26246897

[9] Douedi S, Alshami A, Ashforth G, Maludum O, Carson M. Severe symptomatic bradycardia after intravenous immunoglobulin infusion: a rare manifestation. Journal of Cardiology and Cardiovascular Sciences 2020; 4: 14–17.

[10] Ko Ko NL, Minaskeian N, El Masry HZ. A case of irreversible bradycardia after rituximab therapy for diffuse large B-cell lymphoma. Cardiooncology 2020; 6: 22. 33062307 10.1186/s40959-020-00077-5 PMC7552504

[11] Gearges C, Haider H, Rana V, Asghar Z, Kewalramani A, Kuschner Z. Bebtelovimab-induced bradycardia leading to cardiac arrest. Crit Care Explor 2022; 4: e0747. 36003828 10.1097/CCE.0000000000000747 PMC9394688

[12] Mori Y, Kudo S, Wakamura K, Matsudaira S, Misawa M, Sugihara Y, Miyachi H, Katagiri A, Baba T. Prolonged bradycardia induced by bevacizumab-associated infusion reaction in advanced metastatic colon cancer. Journal of Medical Cases 2015; 6: 194–197.

[13] Dougherty L, Lewis WE, O’Neill M, Bhaumik A, D’Andrea D, Johnson AL. Infusion-related reaction management with amivantamab for EGFR exon 20 insertion mutation NSCLC: a practical guide for advanced practitioners. J Adv Pr Oncol 2024; 15: 245–252. 10.6004/jadpro.2024.15.4.2 PMC11409773 39301102

[14] Rombouts MD, Swart EL, VAN DEN Eertwegh AJM, Crul M. Systematic review on infusion reactions to and infusion rate of monoclonal antibodies used in cancer treatment. Anticancer Res 2020; 40: 1201–1218. 32132017 10.21873/anticanres.14062

